# Peroxisome Proliferator-Activated Receptor Signaling-Mediated 13-S-Hydroxyoctadecenoic Acid Is Involved in Lipid Metabolic Disorder and Oxidative Stress in the Liver of Freshwater Drum, *Aplodinotus grunniens*

**DOI:** 10.3390/antiox12081615

**Published:** 2023-08-15

**Authors:** Miaomiao Xue, Pao Xu, Haibo Wen, Jianxiang Chen, Qingyong Wang, Jiyan He, Changchang He, Changxin Kong, Changyou Song, Hongxia Li

**Affiliations:** 1 Wuxi Fisheries College, Nanjing Agricultural University, Wuxi 214081, China; 2021113016@stu.njau.edu.cn (M.X.); xup@ffrc.cn (P.X.); wenhb@ffrc.cn (H.W.); 2020113020@stu.njau.edu.cn (J.C.); 2022813030@stu.njau.edu.cn (Q.W.); 2022813053@stu.njau.edu.cn (J.H.); 2022813041@stu.njau.edu.cn (C.H.); kcx2023813039@stu.njau.edu.cn (C.K.); 2 Key Laboratory of Freshwater Fisheries and Germplasm Resources Utilization, Ministry of Agriculture and Rural Affairs, Freshwater Fisheries Research Center, Chinese Academy of Fishery Sciences, Wuxi 214081, China

**Keywords:** *Aplodinotus grunniens*, high-fat diet, starvation stress, lipid deposition, oxidative stress, *PPAR* signaling

## Abstract

The appropriate level of dietary lipids is essential for the nutrient requirements, rapid growth, and health maintenance of aquatic animals, while excessive dietary lipid intake will lead to lipid deposition and affect fish health. However, the symptoms of excessive lipid deposition in the liver of freshwater drums (*Aplodinotus grunniens*) remain unclear. In this study, a 4-month rearing experiment feeding with high-fat diets and a 6-week starvation stress experiment were conducted to evaluate the physiological alteration and underlying mechanism associated with lipid deposition in the liver of *A. grunniens*. From the results, high-fat-diet-induced lipid deposition was associated with increased condition factor (CF), viscerosomatic index (VSI), and hepatosomatic index (HSI). Meanwhile, lipid deposition led to physiological and metabolic disorders, inhibited antioxidant capacity, and exacerbated the burden of lipid metabolism. Lipid deposition promoted fatty acid synthesis but suppressed catabolism. Specifically, the transcriptome and metabolome showed significant enrichment of lipid metabolism and antioxidant pathways. In addition, the interaction analysis suggested that peroxisome proliferator-activated receptor (*PPAR*)-mediated 13-S-hydroxyoctadecenoic acid (13 (s)-HODE) could serve as the key target in regulating lipid metabolism and oxidative stress during lipid deposition in *A. grunniens*. Inversely, with a lipid intake restriction experiment, *PPARs* were confirmed to regulate lipid expenditure and physiological homeostasis in *A. grunniens*. These results uncover the molecular basis of and provide specific molecular targets for fatty liver control and prevention, which are of great importance for the sustainable development of *A. grunniens*.

## 1. Introduction

Carbohydrates, lipids, and proteins are the main nutrients for fish to attain essential energy and maintain normal life activities. It is well known that protein-source nutrients—especially the most desirable protein, fish meal—are expensive and subject to a shortage of supply all over the world [1]. Carbohydrates are essential in commercial fish feed formulations as an energy-producing nutrient [2]. However, the nutritional value of dietary carbohydrates for fish varies [3]. Compared to proteins and carbohydrates, lipids contain relatively more energy per unit mass. Lipids are essential for the growth and reproduction of fish and have been highlighted as providing essential fatty acids and facilitate the absorption of fat-soluble vitamins [4]. Therefore, dietary lipids are important for fish to obtain sufficient energy [5,6]. As a non-protein energy substitute, the appropriate addition of lipids in the diet can reduce protein consumption and improve the growth performance and feed conversion of fish [7,8]. Insufficient or deficient dietary lipid content in the diets of fish can cause metabolic disorders and reduced protein utilization and lead to deficiencies in fat-soluble vitamins and fatty acids [9]. However, excessive lipid levels trigger severe lipid accumulation in the liver, lead to metabolic disorders, and inhibit fish growth [10,11,12]. Moreover, excessive lipid accumulation induces reactive oxygen species (ROS), inflammation, and oxidative stress, which negatively affect the growth, physiological homeostasis, and health of aquatic animals [13,14,15].

Lipid accumulation in the liver is caused by a variety of factors. Studies have shown that excessive energy intake, essential nutrient deficiencies, exogenous and endogenous peroxidation, environmental pollution, physiology, and species are important determinants of lipid accumulation in the liver [16]. Excessive lipid intake is the main cause of lipid accumulation in the livers of most fish. One possible reason is that an increase in dietary lipids results in altered lipid metabolism, affecting lipogenesis and fatty acid oxidation [17]. Another important reason is that the liver, as an important central organ for controlling lipid homeostasis [18], oxidizes lipids and also encapsulates excess lipids, secretes them into other tissues (such as fat), and stores them [19]. In addition, once large amounts of fatty acids from lipolysis enter the liver, they form triglycerides and are stored in the liver as lipid droplets [20]. The adverse effects of a high-fat diet have been extensively studied in aquatic animals. High-fat diets affected β-oxidation in the liver of *Megalobrama amblycephala* and led to mitochondrial dysfunction, which subsequently mediated oxidative stress, reduced immunity, and apoptosis [21]. High-fat diets led to lipid metabolism disturbances and reduced immune capacity in *Ctenopharyngodon idella* [22,23], and in *Acanthopagrus schlegelii* and Scophthalmus maximus, these were found to lead to oxidative stress and lipid peroxidation [24,25]. In addition, oxidative damage, apoptosis, and inflammation of the liver have been found in *Oreochromis niloticu* [26]. Furthermore, lipid deposition due to high-fat diets has also been studied in a variety of aquatic animals, such as *Trachinotus ovatus* [27], *Hybrid yellow catfish* [28], and *Lateolabrax japonicus* [13]. However, as a newly domesticated aquatic animal, the effects of high-fat diets in freshwater drum have not been investigated. In addition, the key molecules or signaling pathways involved in lipid metabolism are not well elucidated.

An effective antioxidant system is essential for fish to resist disturbances by adverse external factors [29]. Peroxisome proliferator-activated receptors (*PPARs*), as important nuclear receptors [30], play a key role in the regulation of lipid metabolism, cell growth, inflammation, and differentiation [31]. Many studies have demonstrated that *PPARs* are associated with oxidative stress response. *PPARs* not only directly regulate the expression of pro-oxidant and antioxidant genes but also influence antioxidant and anti-inflammatory responses that interact with other pathways [32]. Different isoforms (*pparα*, *pparβ/δ*, *pparγ*) control various intracellular metabolic processes [33]. In particular, *pparα* is involved in the expression of lipid metabolism [34], and its ligands can activate antioxidant enzymes [35,36]. *Pparβ/δ* is implicated in lipid oxidation and cell proliferation [37], and its activation inhibits ROS production and prevents apoptosis [38]. *Pparγ* is associated with adipocyte differentiation [39] and also promotes oxidative phosphorylation, antioxidant defense, and mitochondrial biogenesis [40]. In addition, linoleic acid oxidative metabolites are natural ligands of *PPARs* [41]. As one of the metabolites, 13-S-hydroxyoctadecenoic acid (13 (s)-HODE) has been found to be an agonist of *pparβ/δ* [42] and can activate *pparγ* [43]. Therefore, this study investigated the regulatory mechanism between liver metabolites and *PPARs* when lipid intake is excessive or limited.

Freshwater drum (*Aplodinotus grunniens*) is a fish endemic to North and Central America. It is the only species in the genus of *Aplodinotus* that perpetually inhabits freshwater [44]. Freshwater drum is characterized by its possession of a higher proportion of edible parts, with delicious and nutritious flesh rich in proteins, amino acids, and fatty acids, especially the unsaturated fatty acids DHA and EPA. Moreover, freshwater drum has no intermuscular bones, which improves the fish quality and the processing of the aquatic product. Therefore, these distinct characteristics reveal that freshwater drum has the potential for domestication and cultivation, providing high-quality proteins for human beings. In light of these prospects, we imported freshwater drum larvae and achieved a worldwide breakthrough in advanced research on artificial breeding, feeding, and domestication. In 2022, we achieved large-scale fry breeding, laying the groundwork for the industrialized development of freshwater drum culture and the breeding of new varieties [45,46,47,48,49]. However, in the practical aquaculture, we found freshwater drum was susceptive to lipid deposition in the liver, which adversely affected the health of the freshwater drum. Therefore, we conducted the current study to evaluate the effects of lipid deposition on physiological and metabolic homeostasis and the molecular mechanisms of its regulation in freshwater drum. Specifically, we evaluated the effects of lipid deposition on the growth performance, physiological homeostasis, and metabolic capacity of freshwater drum. Meanwhile, the interaction between the liver transcriptome and metabolome was also investigated. In addition, the restriction of lipid intake in a starvation stress experiment was used to further reveal the regulatory mechanism in freshwater drum. These findings indicate the molecular basis of liver lipid deposition and provide specific molecular targets for the control and prevention of lipid deposition, which have important implications for the sustainability of freshwater drum. 

## 2. Materials and Methods

### 2.1. Ethics Statement

The study was approved by the Animal Care and Use Committee of Nanjing Agricultural University (Nanjing, China, WXFC 2021-0006). All animal procedures were carried out in accordance with the Guideline for the Care and Use of Laboratory Animals in China.

### 2.2. Experimental Animals and Experimental Design

Experiments were conducted in the breeding base of the Freshwater Fisheries Research Center, Chinese Academy of Fishery Sciences. Approximately 40,000 healthy freshwater drums were randomly transferred to two outdoor fish ponds (pond size: 667 m^2^, 20,000 fish per pond representing three biological replicates) for a high-fat-diet experiment. The control (CL) group and the high-fat-diet (FL) group were fed compound diets four times a day for four months during the experiment (Table S1). The daily feeding amount was 3~5% of the body weight. For the starvation stress experiment, 240 freshwater drums with an average weight of 20.88 ± 2.75 g were randomly assigned into 12 tanks (3 tanks per group, 20 individuals per tank) in indoor temperature-adjustable circular aquaculture systems (specifications for φ: 820 mm × 700 mm, 300 L). In the experiment, starvation for 0 d was set as the control group (Con), and 1 d (Sta1d), 2 w (Sta2w), and 6 w (Sta6w) were set as the starvation treatments. During the experiment, all fish were not fed. The water was obtained from underground with absolute aeration and the temperature was maintained at (26 ± 1) °C. Throughout the experiments, water parameters were kept as follows: dissolved oxygen > 6 mg L^−1^, pH 7.2~7.8, NO_2_^−^ < 0.02 mg L^−1^, and NH_3_ < 0.05 mg L^−1^.

### 2.3. Sample Collection

After 4 months of rearing experiments, fish were starved for 24 h to evacuate the alimentary tract contents. Fish from each group were randomly selected and anesthetized with MS-222 (100 mg/L) to collect samples. For each fish, the final body weight, body length, visceral weight, and liver weight were measured to access the growth performance in terms of the condition factor (CF), viscerosomatic index (VSI), and hepatosomatic index (HSI). After that, the fish were dissected and the liver tissues were collected on ice, immediately frozen in liquid nitrogen, and stored at −80 °C for subsequent analysis. Growth performance was calculated with the following equations:

CF = weight/length^3^ × 100%; VSI = visceral weight/body weight × 100%; HSI = liver weight/body weight × 100%.

### 2.4. Biochemical Index Measurement

Enzyme activity/levels were measured with 10% liver homogenate supernatant according to the manufacturer’s instructions. Specifically, nine liver samples were selected from each group and measured in duplicate. For each liver sample, 0.1–0.2 g were separated, rinsed in ice-cold saline to remove the blood, dried on filter paper, and homogenized in ninefold physiological saline (*w*/*v*). After centrifugation (2500× *g*, 4 °C) for 10 min, the supernatant was collected for further measurement.

Enzymatic activities/levels, including superoxide dismutase (SOD), glutathione peroxidase (GSH-PX), glutathione (GSH), glutamate pyruvic transaminase (GPT), glutamic oxaloacetic transaminase (GOT), total antioxidant capacity (T-AOC), total cholesterol (T-CHO), and triglycerides (TGs), were measured. In detail, SOD was measured with the WST-1 method (Category No. A001-3), GSH-PX with the colorimetric method (Category No. A005-1-2), GSH with the microplate method (Category No. A006-2-1), GPT with the microplate method (Category No. C009-2-1), GOT with the microplate method (Category No. C010-2-1), T-AOC with the colorimetric method (Category No. A015-1), T-CHO with the microplate method (Category No. A111-1-1), and TGs with the microplate method (Category No. A110-1-1). All the assay kits were purchased from Nanjing Jiancheng Bioengineering Institute, Nanjing, China.

### 2.5. RNA Extraction and De Novo High-throughput Sequencing

We used the method described by Song [49]. In the high-fat-diet feeding experiment, 12 fish were selected from each group and three tissues were randomly mixed, so a total of four samples were used for transcriptome sequencing. In the starvation stress experiment, nine fish were selected from each group and three tissues were randomly mixed, so a total of three samples were used for transcriptome sequencing. The first step was cDNA library construction and de novo sequencing. Briefly, RNA quality was examined using Agilent 2100 and Nanodrop (ThermoFisher Ltd., Waltham, MA, USA), and high-quality RNA (1.8 < OD260/280 < 2.0, RNA integrity number (RIN) ≥ 1.8, 28S/18S ≥ 1.0) was treated with oligo (dT) to enrich mRNA. Next, the mRNA was randomly split into small fragments of about 300 bp using random primers. The cDNA was synthesized using short fragments as templates and PCR amplification was performed after the addition of the “A” tail and sequencing connectors. Finally, de novo high-throughput sequencing was undertaken with an Illumina NovaSeq6000 (Majorbio Bio-pharm Technology Co., Ltd., Shang, China). The raw data were filtered to obtain high-grade quality control data. Assembly and splicing of reads sequences were undertaken using Trinity and functional annotation in the NR, Swiss Port, Pfam, KOG, and GO databases. Transcripts with |log_2_fold change| > 1 and corrected *p*-value < 0.05 were considered as differentially expressed genes (DEGs). DEGs were also analyzed for GO and KEGG enrichment.

### 2.6. Metabolome Sample Processing and LC-MS Detection

LC-MS metabolome analysis was undertaken following the method described by Zhang [50]. Specifically, samples were weighed before extracting metabolites (12 individual livers were selected from each experimental group, and 4 livers were randomly mixed), dry-frozen, and then ground. Metabolites were extracted using 1000 μL precooled mixtures of methanol, acetonitrile, and water (*v*/*v*/*v*, 2:2:1) and then placed for 1 h in ice baths with ultrasonic shaking. The mixture was allowed to settle at −20 °C and treated with the high-throughput tissue crusher Wonbio-96c (Shanghai wanbo biotechnology Co., Ltd., Shanghai, China). Finally, the supernatant was carefully transferred to sample vials for LC-MS/MS analysis (UHPLC-Q Extractive system from Thermo Fisher Scientific, Waltham, MA, USA). A pooled quality control (QC) sample was prepared by mixing equal volumes of all samples to monitor the stability of the analysis. The sample was kept in the 4 °C autosampler for the entire analysis, and the samples were separated with an Agilent 1290 Infinity LC ultra-high-performance liquid chromatography (UHPLC) system using an HILIC column. Data were processed using normalization and removal of variables with relative standard deviations (RSDs) > 30% for QC samples, and log10 was logarithmically processed to obtain the final data matrix for subsequent analysis.

### 2.7. Interaction Analysis

The connected networks were plotted using Cytoscape (Version 3.9.0) to elucidate the relationship between genes and metabolites. To explore the relationship between different DEGs and DEMs, a heat map was plotted and Pearson’s correlation test was performed to further analyze the correlations between key genes and key metabolites. The significance threshold was set as *p* < 0.05.

### 2.8. RT-PCR Analysis

RNAiso Plus reagent (Takara Co., Ltd., Dalian, China) was used to extract total RNA from nine livers in each group in duplicate, and they were incubated with RNase-free DNase (Takara Co., Ltd., Dalian, China) to remove contaminated genomic DNA. The quantity and quality of RNA were assessed with the OD260/280 method and 1.5% agarose gel electrophoresis. Primers (Table 1) for each gene were designed using Primer Premier 5.0 based on the mRNA sequences obtained from an *A. grunniens* liver genome database in our lab. All primers were synthesized by Shanghai Generay Biotechnology Co., Ltd., Shanghai, China. RT-PCR was performed with SYBR Green (Takara, Dalian, China) on a Takara 800 Fast Real-Time PCR system according to the manufacturer’s protocol. β-actin was applied as an internal reference and further calculated using the 2^−ΔΔCT^ method.

### 2.9. Statistical Analysis

In the study, all data were calculated using SPSS software (version 26.0) and are presented as means ± standard deviation (SD). For RNA expression analysis, the 2^−∆∆CT^ method was applied. For statistical difference evaluation, data were analyzed with an independent-samples *t*-test when they were normally distributed and homoscedastic; otherwise, a two-independent-samples nonparametric test (Mann–Whitney U) was used, with *p* < 0.05 representing a significant difference. Pearson correlation analysis was performed to evaluate the correlation between genes and metabolites. In general, *p* < 0.05 was considered to be a significant difference or correlation, while *p* < 0.01 was regarded as an extremely significant difference or correlation.

## 3. Results

### 3.1. High-Fat Diet Induced Lipid Deposition and Destroyed Physiological Homeostasis in the Liver

**Figure 1:**
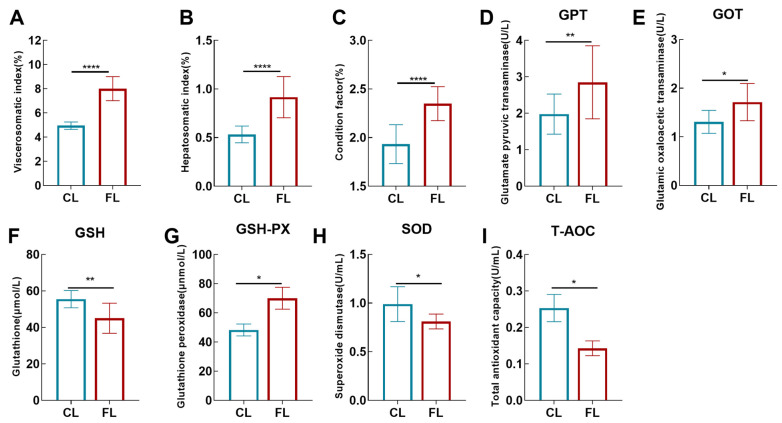
High-fat diet induced lipid deposition and destroyed physiological homeostasis in the liver of *A. grunniens.* (**A**–**C**) Growth performance: (**A**) condition factor (CF); (**B**) viscerosomatic index (VSI); (**C**) hepatosomatic index (HSI). (**D**–**I**) Liver antioxidant enzyme activity: (**D**) glutamate pyruvic transaminase (GPT); (**E**) glutamic oxaloacetic transaminase (GOT); (**F**) glutathione peroxidase (GSH-PX); (**G**) superoxide dismutase (SOD); (**H**) total antioxidant capacity (T-AOC); (**I**) glutathione (GSH). Data were analyzed with Student’s *t*-test. Results are indicated as means ± SD, *n* = 9. * indicates a significant difference between CL and FL groups (*, *p* < 0.05; **, *p* < 0.01; ****, *p* < 0.0001).

Growth performance and antioxidant capacity were first evaluated. The results showed that a high-fat diet resulted in significant increases in the CF, VSI, and HSI (Figure 1A–C, *p* < 0.0001) in freshwater drum compared with the CL group. In addition, GPT (Figure 1D, F (1,16) =11.563, *p* = 0.004, Table S7), GOT (Figure 1E, F (1,13) = 5.370, *p* = 0.037, Table S7), and GSH-PX (Figure 1F, F (1,13) = 7.639, *p* = 0.016) levels were dramatically increased, while SOD (Figure 1G, F (1,15) = 7.541, *p* = 0.015), T-AOC (Figure 1H, F (1,12) = 6.879, *p* = 0.022), and GSH (Figure 1I, F (1,15) = 9.922, *p* = 0.007) levels were markedly decreased in the FL group. These results demonstrated that a high-fat diet improved the growth performance of freshwater drum; however, it also caused physiological disorders and inhibited the antioxidant capacity of freshwater drum.

### 3.2. High-Fat Diet Led to Lipid Metabolism Disorder in the Liver of Freshwater Drum

**Figure 2:**
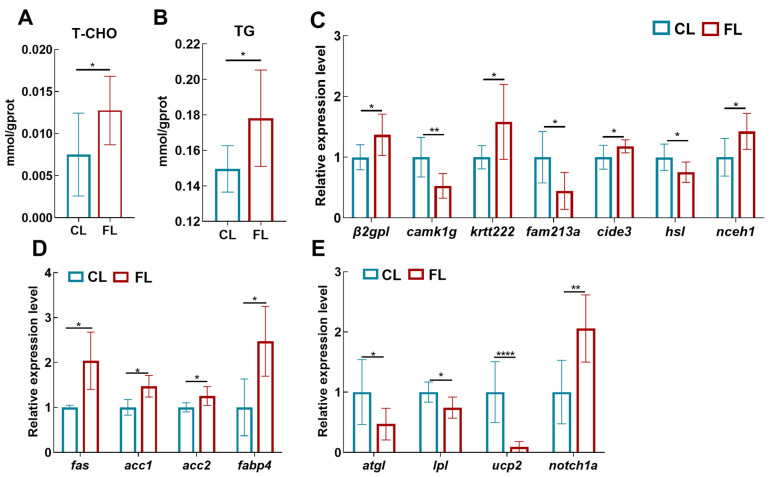
High-fat diet led to lipid metabolism disorder in the liver of *A. grunniens*. (**A**) Total cholesterol (T-CHO); (**B**) triglycerides (TGs); (**C**) expression levels of adipose tissue-specific expressed genes in the fatty liver (*β_2_gp1*, *camk1g*, *krt222*, *fam213a*, *cide3*, *hsl*, *nceh1*); (**D**) fat synthesis-related genes’ expression levels (*fas*, *acc1*, *acc2*, *fabp4*); (**E**) fat lipolysis-related genes’ expression levels (*atgl*, *lpl*, *ucp2*, *notch1a*). Data were analyzed with Student’s *t*-test. Results are indicated as means ± SD, *n* = 9. * indicates a significant difference between CL and FL groups (*, *p* < 0.05; **, *p* < 0.01; ****, *p* < 0.0001).

Based on the above studies, to explore whether physiological disorders caused by a high-fat diet affected the metabolic capacity of the organism, we evaluated the liver fat content and transcriptional expression of lipid metabolism-related genes. The results showed that the contents of T-CHO (Figure 2A, F (1,15) = 5.596, *p* = 0.032) and TG (Figure 2B, F (1,15) = 7.976, *p* = 0.013) in the liver were significantly increased in the FL group. The expression of the adipose-specific genes beta-2-glycoprotein 1-like (*β_2_gp1*, F (1,11) = 6.558, *p* = 0.026), keratin-like protein KRT222 (*krt222*, F (1,11) = 4.871, *p* = 0.049), cell death activator CIDE-3-like (*cide3*, F (1,13) = 5.032, *p* = 0.042), and neutral cholesterol ester hydrolase 1 (*nceh1*, F (1,12) = 6.934, *p* = 0.022) was remarkably upregulated in the FL group (Figure 2C). Meanwhile, we found that the expression of fatty acid synthase (*fas,* F (1,9) = 10.496, *p* = 0.010), acetyl-CoA carboxylase 1 and 2 (*acc1*, F (1,9) = 7.125, *p* = 0.026; *acc2*, F (1,9) = 6.923, *p* = 0.027), and fatty acid binding protein 4 (*fabp4*, F (1,10) = 5.439, *p* = 0.042) was significantly upregulated (Figure 2D). However, the expression of adipose triglyceride lipase (*atgl*, F (1,13) = 6.091, *p* = 0.028), lipoprotein lipase (*lpl*, F (1,9) = 6.145, *p* = 0.035), and uncoupling protein 2 (*ucp2,* F (1,14) = 24.836, *p* = 6.34 × 10^−5^) was markedly downregulated (Figure 2E). These findings demonstrated that lipid deposition led to lipid metabolism disturbances in the liver.

### 3.3. Transcriptomic Analysis Revealed Lipid Metabolism and Antioxidant Pathways Are Significantly Enriched in Livers with Lipid Deposition

**Figure 3:**
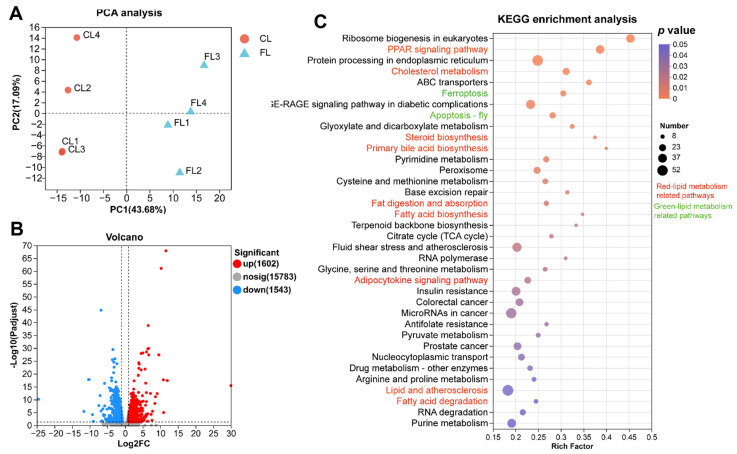
Transcriptomic analysis revealed that lipid metabolism and antioxidant pathways are significantly enriched in livers with lipid deposition. (**A**) PCA analysis of samples; (**B**) volcano plots of expression difference; (**C**) KEGG enrichment of differentially expressed genes: FL group vs. CL group.

To reveal the underlying mechanisms of lipid deposition, we conducted de novo transcriptomic analysis using high-throughput sequencing. Principal component analysis (PCA) score plots showed that the samples in the CL and FL groups were clustered separately, revealing clear differences between the transcriptome profiles of the CL and FL groups (Figure 3A). When compared to the CL group, a total of 3144 differentially expressed genes (DEGs) were identified and annotated in the FL group (log_2_|fold change| > 1 and *p*-value < 0.05), including 1602 upregulated and 1542 downregulated genes (Figure 3B). To reveal the molecular functions of these DEGs, GO and KEGG enrichment analyses were performed. The results indicated that a total of 62 GO items (Table S2) and 36 KEGG pathways (Table S3) were enriched. Specifically, lipid metabolism (*PPAR* signaling pathway, cholesterol metabolism, steroid biosynthesis, primary bile acid biosynthesis, fat digestion and absorption, fatty acid biosynthesis, adipocytokine signaling pathway, lipids and atherosclerosis, fatty acid degradation), glucose metabolism (insulin resistance, pyruvate metabolism), amino acid metabolism (cysteine and methionine metabolism; glycine, serine, and threonine metabolism; arginine and proline metabolism), and oxidative stress (peroxisome, ferroptosis, apoptosis-fly) were the most significantly enriched KEGG pathways (Figure 3C), revealing that lipid deposition affected lipid metabolism, glucose metabolism, amino acid metabolism, and antioxidant capacity.

### 3.4. Metabolomic Analysis Revealed Lipid Metabolism and Antioxidant Pathways Are Significantly Enriched in Livers with Lipid Deposition

**Figure 4:**
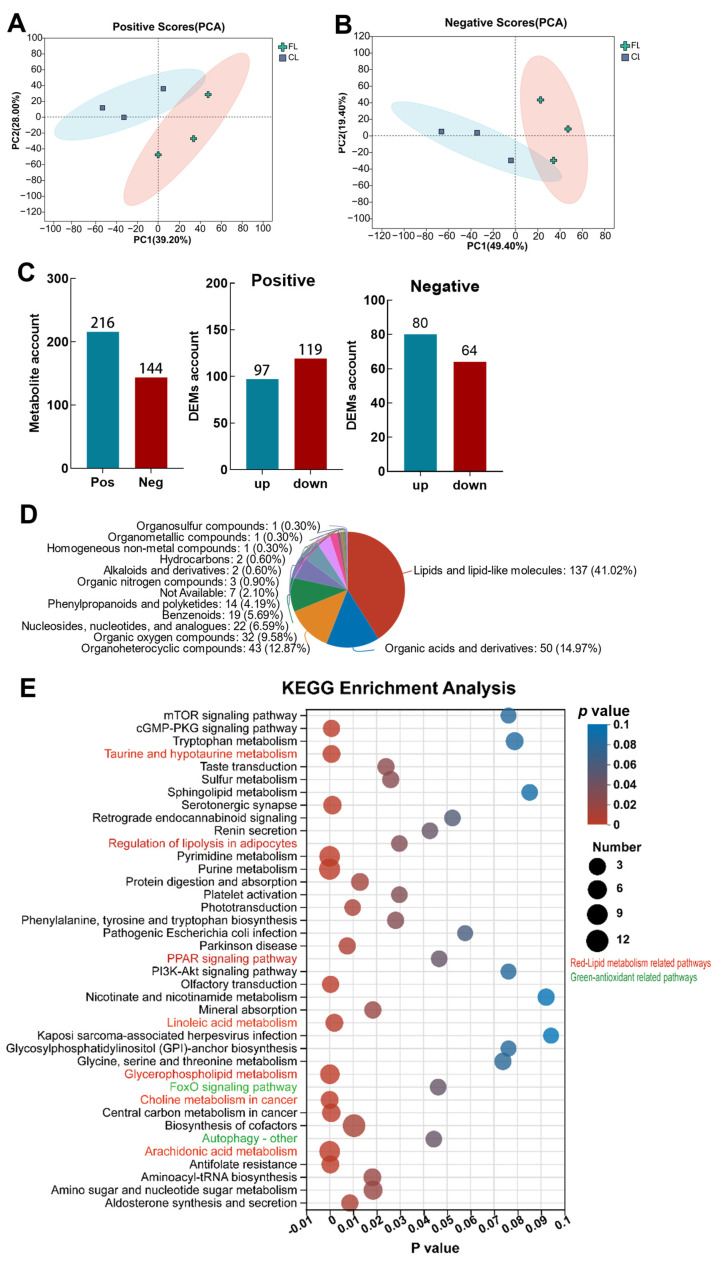
Metabolomic analysis revealed that lipid metabolism and antioxidant pathways are significantly enriched in livers with lipid deposition. (**A**,**B**) PCA analysis of samples: (**A**) positive, (**B**) negative; (**C**) differential metabolite statistics; (**D**) classification of HMDB compounds; (**E**) enrichment analysis of KEGG pathway.

In order to further explore the potential regulatory mechanism, LC-MS-based metabolomics was used to determine the changes in metabolites. The differential metabolites were screened with |log_2_ FC| > 1, *p* ≤ 0.05, and VIP ≥ 1. The principal component analysis (PCA) score plots showed that positive and negative ions in the CL and FL groups were clustered into different subclasses, revealing clear differences between the metabolites of the CL and FL groups (Figure 4A,B). A total of 216 effective positive metabolites and 144 negative metabolites were identified, of which 97 and 80 were upregulated and 119 and 64 were downregulated, respectively (Figure 4C). The classification of HMDB compounds showed that lipids and lipid-like molecules occupied the largest proportion (41.02%, Figure 4D). Additionally, lipid metabolism (regulation of lipolysis in adipocytes (map04923), taurine and hypotaurine metabolism (map0043), the *PPAR* signaling pathway (map03320), linoleic acid metabolism (map00591), glycerophospholipid metabolism (map00564), choline metabolism in cancer (map05231), and arachidonic acid metabolism (map00590)), autophagy (map04136), and *FOXO* pathway signals (map04068) were significantly enriched (Table S4 and Figure 4E) in KEGG pathways. These results provided further evidence that lipid deposition was the dominant driver for lipid metabolism disorder; moreover, lipid deposition affected the regulation of autophagy and redox processes in the liver of freshwater drum.

### 3.5. DEM and DEG Interaction Analysis Revealed that PPAR Signaling Is Involved in the Regulation of Lipid Deposition

**Figure 5:**
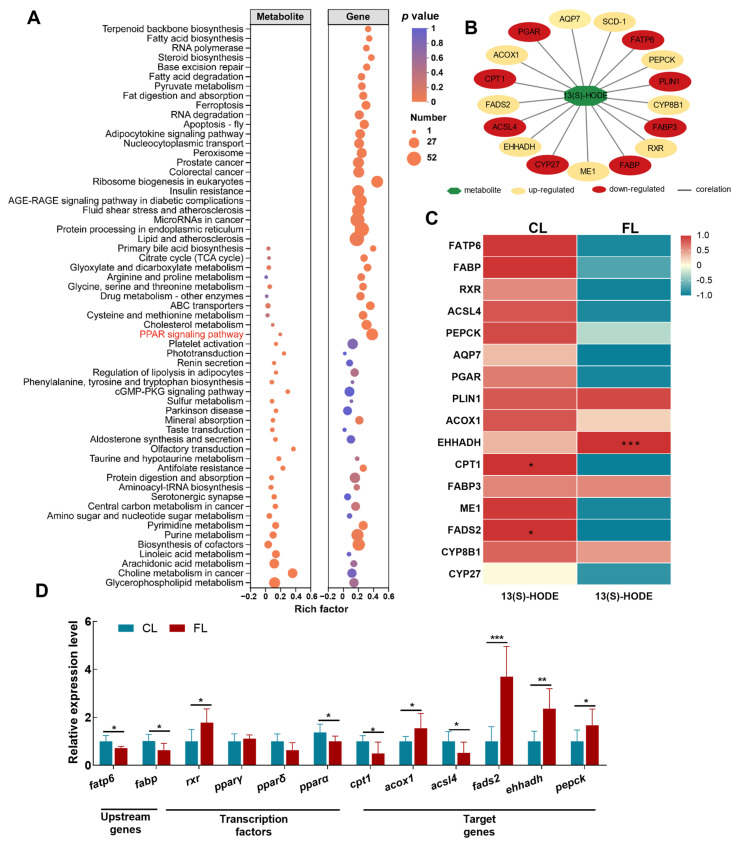
DEM and DEG interaction analysis revealed that *PPAR* signaling is involved in the regulation of lipid deposition. (**A**) KEGG enrichment analysis of transcriptome and metabolome. (**B**) regulatory networks of key enriched genes and metabolite interactions in the *PPAR* signaling pathway. In the network, nodes in yellow represent the upregulated DEGs, those in red represent the upregulated DEGs, and those in green represent the microbe. (**C**) Pearson correlation analysis of key genes and metabolites in the *PPAR* signaling pathway. (**D**) Validation of key gene expression in *PPAR* signaling. Data were analyzed with Student’s *t*-test. Results are indicated as means ± SD, *n* = 9. * indicates a significant difference between the CL and FL groups (*, *p* < 0.05; **, *p* < 0.01; ***, *p* < 0.001).

Next, the correlation between DEGs and DEMs was analyzed to reveal the underlying mechanisms involved in lipid metabolic disorder under lipid deposition. KEGG enrichment analysis demonstrated that *PPAR* signaling is a critical part of coping with lipid deposition (Table S5 and Figure 5A). Based on targeting analysis, activation of *PPAR* caused accumulation of the differential metabolite 13 (s)-HODE (Figure 5B). The correlation analysis determined that key genes in *PPAR* were significantly correlated with 13 (s)-HODE. Specifically, carnitine palmitoyl transferase 1 (*cpt1*, F (1,9) = 5.761, *p* = 0.040) and fatty acid desaturase 2 (*fads2*, F (1,12) = 28.251, *p* = 0.0001) were remarkably correlated in the CL group, and enoyl-coenzyme A hydratase/3-hydroxy acyl-coenzyme A dehydrogenase (*ehhadh*, F (1,14) =17.046, *p* = 0.001) showed an extremely significant correlation in the FL group (Figure 5C). In addition, we found that the relationship between genes and metabolites significantly enriched in *PPAR* signaling was that upstream genes (fatty acid transport protein 6 (*fatp6*), fatty acid-binding protein (*fabp*)) regulated the expression of downstream target genes (such as *cpt1*, acyl-CoA oxidase 1 (*acox1*), acyl-CoA synthetase long-chain family member 4 (*acsl4*), *fads2*, *ehhadh*, phosphoenolpyruvate carboxykinase (*pepck*), etc.) through eicosanoid (13 (s)-HODE)-activated transcription factors (*rxr*) (Figure 5D). The above findings indicated that *PPAR*-mediated 13 (s)-HODE may be a key target of lipid metabolism and physiological homeostasis during lipid deposition in *A. grunniens.*

### 3.6. Transcriptomic Analysis Revealed that PPAR Signaling Is Involved in Regulation of Lipid Consumption

**Figure 6:**
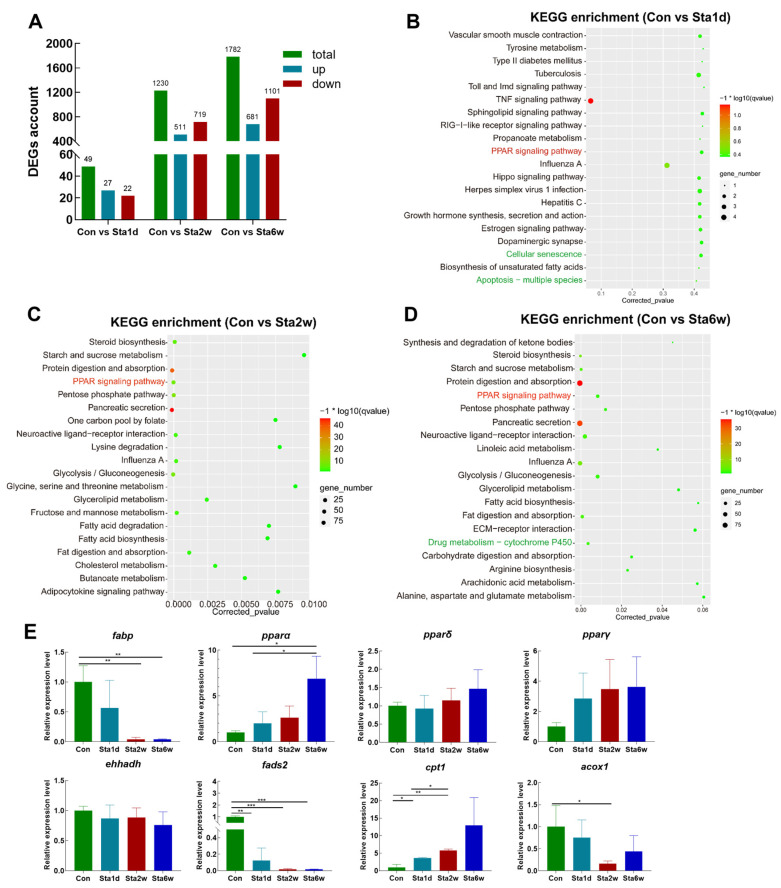
Transcriptomic analysis revealed that *PPAR* signaling is involved in regulation of lipid consumption and inflammatory responses in *A. grunniens*. (**A**) Account of differentially expressed genes at different starvation times; (**B–D**) KEGG enrichment of differentially expressed genes at different starvation times: (**B**) control vs. 1 d starvation, (**C**) control vs. 2 w starvation, (**D**) control vs. 6 w starvation; (**E**) validation of key gene expression. Data were analyzed with Student’s *t*-test. Results are indicated as means ± SD, *n* = 9. * indicates a significant difference between Con and different starvation times (*, *p* < 0.05; **, *p* < 0.01; ***, *p* < 0.001).

To identify potential regulatory mechanisms in the liver after lipid intake restriction in freshwater drum, high-throughput sequencing was performed with liver tissue from the starvation stress experiment. With the analysis of transcriptome sequencing, a total of 49, 1230, and 1782 DEGs were identified in Sta1d, Sta2w, and Sta6w, respectively, of which 27, 511, and 681 were upregulated and 22, 719, and 1101 were downregulated, respectively (Figure 6A). In order to reveal the molecular function of these DEGs, KEGG enrichment analysis was performed and *PPAR* signals were significantly enriched at different starvation times (Table S6 and Figure 6B–D). In addition, we also found that the cellular senescence and “apoptosis-multiple species” pathways were enriched with 1 d starvation, and “drug metabolism-cytochrome *P450*” was enriched with 6 w starvation. RT-PCR validation of key genes in *PPAR* signaling revealed that starvation stress increased the expression levels of *pparα*, *pparδ*, *pparγ*, and *cpt1* and decreased *fabp*, *acox1*, *fads2*, and *ehhadh* (Figure 6E). The above findings demonstrate that *PPARs* can regulate lipid consumption and short-term starvation stress can alleviate oxidative stress in freshwater drum.

### 3.7. Hypothesized Regulatory Mechanisms of Freshwater Drum

**Figure 7:**
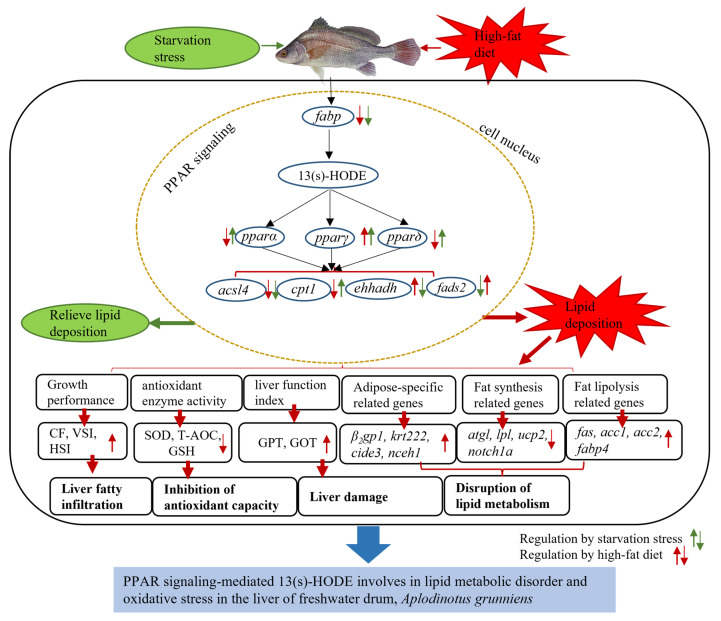
Hypothesized regulatory mechanisms of *A. grunniens.* Hypothetical mechanisms for the regulation of lipid deposition in relation to physiological homeostasis and lipid metabolism, as well as *PPAR* signaling, are proposed based on the results of this study. Green and red arrows indicate positive and negative regulation of genes in the starvation stress experiment and the high-fat-diet experiment, respectively.

According to the above study, we provide a possible schematic representation of the regulatory mechanisms of lipid deposition (Figure 7). Through transcriptome analysis of starvation stress and high-fat-diet experiments, we found that the expression of key genes *paprα*, *pparδ*, and *pparγ* in *PPAR* signaling was upregulated when lipid intake was restricted, and the downstream target genes *cpt1*, *ehhadh*, and *fads2*, which were significantly associated with the metabolite 13 (s)-HODE, showed an opposite expression trend to that of the high-fat diet. These results demonstrate that starvation stress can regulate lipid consumption in freshwater drum through *PPARs*. In addition, our results demonstrate that the disturbance of *PPARs* and metabolites caused by excessive lipid intake can cause oxidative stress and disrupt the physiological homeostasis and lipid metabolic balance of freshwater drum. The results of this study illustrate the hazards of excessive lipid intake in freshwater drum and demonstrate that *PPAR* signaling and 13 (s)-HODE are key molecular targets in regulating lipid metabolism and oxidative stress. These results provide new insights into the potential regulatory mechanisms of freshwater drum under a high-fat diet.

## 4. Discussion

A high-fat diet leads to excessive lipid deposition in the liver [51], resulting in structural damage and metabolic disturbances, which ultimately affect the growth and health of fish [52]. In the present study, the CF, HSI, and VSI were remarkably increased after intake of the high-fat diet, indicating that the high-fat diet induced nutrient deposition in the fish, which was in accordance with findings for *T. ovatus* [27], *Oncorhynchus mykiss* [53], and *Rachycentron canadum* [12]. However, a high-fat diet induces significant increases in T-CHO and TG levels in the liver. Studies have shown that higher levels of dietary soybean oil addition are associated with increased liver lipid deposition in *S. maximus* [54], and liver fat accumulation and tissue abnormalities were found in *Megalobrama amblycephala* on a high-fat diet [55]. This same result was also observed in *O. niloticus* [56]. From the point of view of energy utilization, a possible explanation is that the high-fat diet provides more energy than the growth requirements of freshwater drum, thus leading to metabolic disequilibrium. Moreover, alterations in liver fat content can provide strong evidence for an in-depth study of metabolic disorders.

High-fat diets tend to cause lipid accumulation and peroxidation in fish livers [57,58]. Cells respond to damage caused by lipid accumulation and peroxidation by producing reactive oxygen species (ROS), but ROS have a strong oxidative capacity [59]. They can cause hepatocyte dysfunction by damaging intracellular macromolecules (including lipids, proteins, and DNA), and inhibition of ROS can alleviate hepatocyte lipid accumulation [60]. As a peroxidase, GSH-Px can convert peroxides into harmless hydroxyl compounds and water to prevent them from oxidizing and forming dangerous free radicals [61,62]. In our study, we found that liver lipid deposition increased GSH-Px levels, which might have been due to a self-protection mechanism to resist lipid deposition-induced oxidative stress. Specifically, GSH-Px converts harmful peroxides to components without oxidizing properties during this process. However, further studies revealed that SOD, GSH, and T-AOC levels were significantly decreased. The antioxidant enzyme SOD is often considered the first line of defense against oxidative stress [63] and is able to dismount superoxide anions into hydrogen peroxide. GSH performs its action as a scavenger by oxidizing to GSSG [64]. In addition, T-AOC is able to respond to the total antioxidant level representing various antioxidant substances and antioxidant enzymes. These results indicate that fatty deposits reduce the antioxidant capacity of the liver. Furthermore, the elevated levels of GPT and GOT, which reflect the degree of hepatocyte damage, were further evidence of possible liver tissue damage. This has also been studied in *Micropterus salmoides* [65] and *M. amblycephala* [66].

There is evidence that excessive lipid accumulation in liver cells can cause damage to the liver metabolic system [67]. Therefore, we further explored the effect of lipid deposition on the metabolic capacity of freshwater drum. We discovered that the high-fat diet significantly upregulated the expression of adipose-specific genes (*β_2_gp1*, *cide3*, *nceh1*, and *krt222*) and adipogenic genes (*fas, acc1*, *acc2*, and *fabp4*). This is consistent with findings for other fish [68,69,70]. These results demonstrate that the high-fat diet altered the rate of lipid transport, accelerated the process of lipid accumulation, increased the rate of cholesterol metabolism, and enhanced the protection of cellular structures in the face of liver damage [71]. As is known, an imbalance between lipogenesis and lipolysis can lead to abnormal lipid deposition [72]. We observed that the expression levels of key lipolytic genes (*atgl*, *lpl*, and *ucp2*) were decreased, indicating a slowed rate of lipolytic metabolism in the liver. Moreover, it has been shown that activation of notch signaling in hepatocytes causes decreased glucose metabolism and adipogenesis, leading to lipid accumulation [73]. This was also verified by the upregulation of notch1a expression. In general, lipid deposition due to a high-fat diet increases fatty acid synthesis and decreases lipolysis, affecting lipid metabolism in the liver and resulting in a disruption of lipid metabolism.

Based on the above studies, the transcriptome and metabolome were used to reveal the response mechanisms of freshwater drum more comprehensively in response to lipid deposition. In this study, we found that DEGs were mainly involved in lipid metabolism, glucose metabolism, and amino acid metabolism, as well as antioxidant processes. DEMs were mainly involved in lipid metabolism, and autophagy and antioxidant defense metabolites were also enriched. Interaction analysis showed that the classical *PPAR* signaling-mediating lipid metabolism is involved in the regulation of liver lipid deposition in freshwater drum. The same finding has been reported for *M. salmoides* [74] and *O. niloticus* [75]. As a regulator, *PPAR* controls the expression of a series of genes involved in lipid and lipoprotein metabolism [76]. In addition, our previous study found that *PPAR* signaling is implicated in the metabolic homeostasis of lipids and amino acids in freshwater drum with hypothermia [48]. *PPARs* are activated under starvation stress to regulate the expression of downstream adipose catabolism genes [77]. These studies illustrated the importance of *PPAR* signaling for the regulation of lipid metabolism homeostasis in freshwater drum. Furthermore, the expression levels of all three isoforms showed a trend for upregulation in the starvation stress experiment. It has been shown that *pparα* activation promotes lipolysis metabolism during fasting [78], which is consistent with the results of the present study. In addition, *pparδ* has been shown to have a similar metabolic effect to *pparα* in promoting energy dissipation and, conversely, *pparγ* promotes energy storage [79]. Therefore, it can be inferred that, when the intake of exogenous lipids is limited, freshwater drum will not only decompose fat to provide energy for the organism but also store energy to ensure normal life activities. In addition to the abovementioned functions of *PPARs* in regulating lipid metabolism, as multifunctional nuclear receptors, *PPARs* also play vital roles in regulating physiological homeostasis and responding to external stresses. In this study, we found that high-fat diets can cause damage to the antioxidant system of the liver. However, when starved for 2 w, cell fate, apoptosis, and antioxidant processes were not affected, indicating that appropriate starvation may enhance the anti-stress ability of freshwater drum, which further proves the strong adaptability of freshwater drum to the external environment.

Transcriptome and metabolome interaction analysis revealed that the key metabolite 13 (s)-HODE was significantly correlated with *ehhadh*, *fads2*, and *cpt1*. *ehhadh* and *cpt1* are involved in fatty acid β-oxidation [80,81], and *fads2* is associated with fatty acid metabolism and adipose tissue inflammation [82,83]. Studies have shown that 13 (s)-HODE can increase lipid uptake, return cholesterol transport, and apoptosis. As a marker of oxidative stress, high-fat diets also lead to increased 13 (s)-HODE. It was hypothesized that the increase in 13 (s)-HODE due to lipid deposition caused by a high-fat diet could, in turn, further exacerbate lipid deposition and oxidative stress. However, in the starvation stress experiment, we found that the key genes *cpt1*, *ehhadh*, and *fads2* showed different trends compared to the liver with lipid deposition. This not only indicates that lipid deposition affects liver lipid metabolism, stress resistance, and physiological homeostasis but also demonstrates that *PPARs* can regulate lipid consumption and reduce oxidative stress caused by lipid deposition in freshwater drum when lipid intake is restricted. In accordance with the above findings, *PPAR* signaling-mediated 13 (s)-HODE was identified as a key target for lipid metabolism and oxidative stress regulation in the liver of freshwater drum.

## 5. Conclusions

In summary, the current study suggests that lipid deposition in the liver caused by a high-fat diet leads to physiological disorders and affects liver metabolic capacity. Moreover, transcriptome and metabolome studies revealed that *PPAR* signaling and metabolites (13 (s)-HODE) might be the key targets for regulating liver lipid deposition and oxidative stress in freshwater drum. In addition, *PPARs* can regulate lipid consumption and enhance stress capacity in freshwater drum during periods of lipid intake restriction. These results reveal potential regulatory mechanisms in the liver of freshwater drum with lipid deposition.

## Figures and Tables

**Table 1:** Primer sequences for target genes for RT-PCR analysis.

Accession No.	Gene	Primer Sequence (5′—3′)
XP_008328442.1	*β-actin*	F: AGGCTGTGCTGTCCCTGTAT R: GCTGTGGTGGTGAAGGAGTAG
XP_010742647.2	*β_2_gp1*	F: GGCAGTATCCTCACCCCATC R: CCTTCTGAGGTCCATCCAGC
KKF21127.1	*camk1g*	F: TACATGCTCGGCTCCACTCT R: TCTCCTTCACGCTCAACTCG
KKF23363.1	*krt222*	F: GAGAGTGCAGAAGGTCACGG R: GGGGAGGCTGTCCTGTTTAG
XP_018535573.1	*cide3*	F: ACCCCACATCCAAACAGCAT R: TTTTTGGCAGCGTAACAGCG
XP_019122735.1	*hsl*	F: TTGCTGAGATGAGGGTGGA R: ACAGGCTGGTCTATGTTCC
XP_010730495.2	*nceh1*	F: TATTAACGGTGGCGTTCGCT R: AAAGAAGCCAGGTGCATCGT
XP_010741055.2	*fam213a*	F: CCCGTGAAAGAAAGATGG R: GTCCAATGACGAACACCC
KKF24881.1	*fas*	F: TGGCATCGAGTACAACAAGC R: TTGGCACGAAGTAGCATCAC
XP_019127403.1	*acc1*	F: CTGGAGGAGACGGTGAAAAG R: TGCGTATCTGCTTGAGGATG
XP_018523996.1	*acc2*	F: AGAGGACCATCCGTTTTGTG R: TTCAGAGGAATGACCCCATC
XP_010755203.2	*fabp4*	F: CAGACGGTCGAAAGACCAAG R: TCATGGCAACAACATCATCC
XP_010739642.2	*atgl*	F: ACGGGGAGAACATACTGGTG R: GTGGAAGCTGGTGGAGTTGT
XP_010729007.2	*lpl*	F: CAGCCGTGCAGTATGTGACT R: AGGTTTTGGAGGTGCTGTTG
XP_019132010.1	*ucp2*	F: ATTCGTGGTCTGTGGAAAGG R: CTGCACCAAAGGCTGATAGG
XP_019134682.1	*notch1a*	F: ACTCAAATGGCTCCCTCCTT R: TCAGTTTCCCCATCTCTGCT
XP_019127721.1	*fatp6*	F: TCAGATCCAGCGTGTGTACG G: CAACAAGGCAACGCAGTCTC
XP_027137407.1	*acsl4*	F: GGCACCCGAGATGTACTGAG R: ACTCCGCTCTGGTTTCACAG
KAE8296399.1	*cpt1*	F: TCAGAGGCAGGAGCCCTATT R: GTGCATGTTCACCACGTTCC
XP_010744948.3	*fabp*	F: TGGTGAAAACCCTGAGCACC R: GCACTTGCACCAGTTTGTCT
XP_019127104.1	*rxr*	F: CAAGCTGTTGCTGCGGTTAC R: TCATTTGATGCGGGGCTTCT
XP_010746626.2	*acox1*	F: TTACCAGCGCATCAGTGGAG R: CTGCGTTGGTTGTCCATGTG
AGG69480.1	*fads2*	F: GAAACAGCTTACGCACTCTGC R: AAGTTGCTCTCCATCCACAGG
XP_019126127.1	*ehhadh*	F: CCTGGTCATTGAGGCTGTGT R: GTTACGGGTTTGAGAGGCCA
XP_019131039.1	*pepck*	F: CCACGTCAACTGGTTCAGGA R: CAGCCAGCCGATAATGCTCT
XP_010747326.2	*pparα*	F: GTGCCTCTCTGTGGGAATGT R: GCTTCGTGGATCTGCCTTAC
XP_019110784.1	*pparγ*	F: GCCTTTGTCTGCCTCTCAAC R: GACCTCGCTACCCTTTCCTC
XP_010746753.2	*pparδ*	F: ATCACCGTCGCTGTCAGAAC R: CCCTTACAACCCTCACAGG

Note: The mRNA sequences for each gene were obtained from an *A. grunniens* transcriptome sequencing database that was preserved in the lab.

## Data Availability

The data presented in this study are available in the article and Supplementary Materials.

## Supplementary Materials

The following supporting information can be downloaded at: https://www.mdpi.com/article/10.3390/antiox12081615/s1, Table S1: Formulation and proximate composition of experimental diets; Table S2: Transcriptome GO enrichment analysis statistics for the high-fat-diet experiment; Table S3: Transcriptome KEGG enrichment analysis statistics for the high-fat-diet experiment; Table S4: Metabolome KEGG enrichment analysis statistics for the high-fat-diet experiment; Table S5: KEGG enrichment analysis statistical table for transcriptome and metabolome interaction analysis; Table S6: Transcriptome KEGG enrichment analysis statistics for the starvation stress experiment; Table S7: Standard curves and variation coefficients for GOT and GPT.
